# Cost-Effectiveness of the EdAl (Educació en Alimentació) Program: A Primary School-Based Study to Prevent Childhood Obesity

**DOI:** 10.2188/jea.JE20170111

**Published:** 2018-12-05

**Authors:** Marta Conesa, Elisabet Llauradó, Magaly Aceves-Martins, David Moriña, Oriol de Solà-Morales, Montse Giralt, Lucia Tarro, Rosa Solà

**Affiliations:** 1 Universitat Rovira i Virgili, Facultat de Medicina i Ciències de la Salut, Functional Nutrition, Oxidation, and Cardiovascular Diseases Group (NFOC-Salut), Health Education and Promotion, Reus, Spain; 2 Unit of Infections and Cancer (UNIC - I&I), Cancer Epidemiology Research Program (CERP), Catalan Institute of Oncology (ICO)-IDIBELL, L’Hospitalet de Llobregat, Barcelona, Spain; 3 Institut d’Investigació Sanitaria Pere Virgili (IISPV), Reus, Spain; Health Institute for Technology Transfer (HITT), Barcelona, Spain; 4 Universitat Rovira i Virgili, Facultat de Medicina i Ciències de la Salut, Functional Nutrition, Oxidation, and Cardiovascular Diseases Group (NFOC-Salut), Unit of Pharmacobiology, Health Education and Promotion, Reus, Spain; 5 Universitat Rovira i Virgili, Reus, Spain. Facultat de Medicina i Ciències de la Salut, Functional Nutrition, Oxidation, and Cardiovascular Diseases Group (NFOC-Salut), Health Education and Promotion, Hospital Universitari Sant Joan de Reus, Spain

**Keywords:** cost-effectiveness, school-based intervention, childhood obesity, prevention

## Abstract

**Background:**

The cost-effectiveness of childhood obesity prevention interventions is critical for their sustained implementation. This study evaluated the cost-effectiveness of the Educació en Alimentació (EdAl) program, a school-based intervention for reducing obesity.

**Methods:**

Total EdAl program implementation costs and per-child costs were estimated. Cost-effectiveness, defined using the incremental cost-effectiveness ratio (ICER), was estimated as the difference between the intervention and control group costs divided by the obesity-related outcome effects for boys (avoided cases of obesity, obesity prevalence, body mass index [BMI], and BMI z-score units) for each group. As a significant difference (4.39%) in the reduction of obesity prevalence between the intervention and control groups was observed for boys in the EdAl program, the data were calculated only for boys.

**Results:**

The intervention cost was 24,246.53 € for 1,550 children (15.64 €/child/3 years) or 5.21 €/child/year. The ICERs/boy were 968.66 € to avoid one case of obesity, 3.6 € to reduce the obesity prevalence by 1%, 44.68 € to decrease BMI by one unit, and 65.16 € to reduce the BMI z-score by one unit.

**Conclusions:**

The cost of reducing the obesity prevalence in boys by 4.39% was 5.21 €/child/year, half the cost proposed by the Spanish Health Ministry, indicating that the EdAl program is cost-effective.

## INTRODUCTION

Childhood obesity is one of the greatest health challenges of the 21st century.^1^ In addition to the clinical effectiveness of interventions to reduce obesity,^2^ the cost-effectiveness of childhood obesity prevention interventions should be evaluated before a sustained intervention is implemented.^2^ Such information is used as a criterion to help policymakers and educators allocate resources toward the most appropriate preventive interventions.^3^^,^^4^ Schools have been identified as a key setting for health promotion interventions to prevent childhood obesity.^5^

The primary outcome of cost-effectiveness analysis is the ratio of the net costs of an intervention divided by the net gain in health effects, such as obesity-related outcomes.^3^^,^^4^ Surprisingly, cost-effectiveness analyses for childhood obesity prevention programs are scarce compared with the high number of prevention studies,^2^ and adequate methodologies remain under debate.^3^ However, a group that specializes in assessing the cost-effectiveness of obesity prevention programs has described the important aspects of such analyses.^6^ In Spain, the Health Ministry criteria suggest that a childhood obesity prevention intervention is cost-effective when the costs to reduce the obesity prevalence by more than 2% are less than 5 € per student per year.^5^ These factors suggest the need to conduct a cost analysis of the Educació en Alimentació (EdAl) program after its effectiveness was confirmed. The EdAl program is a randomized, parallel, controlled primary school-based obesity prevention intervention implemented in various cities of Catalonia that encourages healthy lifestyle choices through diet and physical activity recommendations for children aged 7–8 years over a period of 28 months (3 academic years; this study examined the 2007 to 2010 school years). The EdAl program is effective for reducing obesity-related outcomes, such as the obesity prevalence and the body mass index (BMI) z-score.^5^ The program also improves secondary outcomes, such as increasing after-school physical activity among boys in the intervention group compared with the control group.^5^ Consequently, this study aimed to evaluate the cost-effectiveness of the EdAl program.

## METHODS

### EdAl program overview

The EdAl program was administered by undergraduate students in the medical and health sciences who were trained as health promoter agents (HPAs). The HPA coursework was divided into two 45-hour courses taught during the same academic year, for a total of 90 hours/year. These two courses were not compulsory. The coursework focused on the methodological basis for promoting health and on strategies for designing and implementing activities that addressed eight healthy lifestyle topics related to nutrition and physical activity. The EdAl program was approved by the Clinical Research Ethics Committee of the Hospital Universitari Sant Joan of Reus, Universitat Rovira i Virgili (Catalan Ethics Committee Registry #20; ref: 08-07-24/07aclproj1). The protocol conformed to the Helsinki Declaration and the Good Clinical Practice guidelines of the International Council for Harmonization. The protocol of the EdAl program (trial registration number: ISRCTN29247645)^7^ and its results have been published.^5^ In the EdAl program, baseline data were recorded every year for 3 academic years (2007–2010); the data included the students’ name, gender, date and place of birth, and anthropometric measurements (weight, height, BMI, and waist circumference).^7^

This study followed the CHEERS checklist for cost-effectiveness analysis studies (eTable 1).^8^

### EdAl intervention program

The EdAl intervention was based on 12 educational intervention activities over 3 academic years (1 hour/activity/session; four activities/year), which were prepared and standardized by the HPAs and implemented in the children’s classrooms. These activities were focused on the following eight lifestyle topics based on scientific evidence^9^: 1) improving healthy lifestyle choices; 2) encouraging the intake of healthy drinks; 3) increasing the consumption of vegetables and legumes; 4) decreasing the consumption of candy and pastries, while increasing the intake of nuts; 5) improving healthy habits and physical activity within a set timetable; 6) increasing fruit intake; 7) improving dairy product consumption; and 8) increasing fish consumption.^7^ As described previously,^5^^,^^7^ the intervention program comprised three components: 1) classroom practice led by HPAs to emphasize the eight healthy lifestyle habits through 12 educational intervention activities, distributed as four activities/year for three academic years; 2) teaching practices for HPAs that used custom-designed booklets (as teaching aids) or other educational support for the eight lifestyle topics presented in the educational activities; and 3) workshops for parents to build on the activities in which their children participated.

Each classroom-based educational intervention activity also had three components: 1) the experimental development of activities related to healthy lifestyle choices using food (free food was provided by local producers) to allow the children to experience organoleptic qualities that may or may not be new to them; 2) assessments of the knowledge the children gained from each classroom-based activity; and 3) activities developed in the classroom for use at home.

A report on the effectiveness of the EdAl program after 28 months (2.3 years)^5^ revealed that the obesity prevalence, determined using the IOTF definition,^10^ decreased significantly (by 2.02%) in the intervention group and increased by 0.44% in the control group. The obesity prevalence among the boys in the intervention group decreased, by 2.36% (from 9.59% to 7.23%; *P* = 0.155), while the obesity prevalence among the boys in the control group increased by 2.03% (from 7.40% to 9.43%; *P* = 0.390), leading to a significant difference of 4.39% (95% confidence interval (CI), 3.48–5.30%; *P* = 0.01). Moreover, the boys in the intervention group exhibited an effective reduction of −0.24 units in the BMI z-score (from 0.01 to −0.04) compared with the control group (from −0.10 to 0.09). However, the obesity prevalence did not significantly decrease among the girls in the intervention group compared with the girls in the control group.

### Cost analysis of the EdAl intervention program

The total costs of implementing the EdAl intervention program were classified into three main categories, as shown in Table 1 (the costs marked as “1” are required for future administration, and those marked as “0” are not required for future administration): 1) human resources, salaries of the paid staff members (coordinator, management, HPA coursework instructors and anthropometric measurement professionals); 2) materials, resources for activities and for monitoring anthropometric measurements; and 3) additional didactic materials, booklets and support materials for parent activities. The cost of implementing the intervention is derived from an institutional perspective due to included direct costs, personal costs, and logistic costs. In the present study, excluded costs comprised those that were not necessary for the future implementation of the program because they were related to effectiveness reassessment. Such costs included those that covered management and coordination (as HPA training is considered part of the job requirements of university professors); anthropometry professionals (who were needed only for the effectiveness reassessment); travel expenses and meals; materials such as scales, stadiometers, and waist tapes (which were used only for the effectiveness reassessment); and additional materials, such as booklets for the parents and children (considered an optional part of training for the study).

**Table 1.** EdA1^a^ program costs included in the cost-effectiveness evaluation

Category	Concept	2007 (€)	2008 (€)	2009 (€)	2010 (€)	Total cost (€)	Required for future application	Result
**Human resources**	**Salaries**							
	Management (6% dedication of total hours – 127 €/month)	1,524.50	1,524.50	1,524.50	1,524.50	6,098.00	0	0
	Coordinators (Predoctoral fellowship stipulated price)	12,000.00	12,000.00	12,000.00	12,000.00	48,000.00	0	0
	Anthropometric professionals (6 €/hour person)	3,008.00	3,008.00	3,008.00		9,024.00	0	0
	HPAs training (30 €/hour)	3,528.00	3,528.00	3,528.00		10,584.00	1	10,584.00
	**Fungible**							
	Travel expenses and meals for anthropometric professionals (0.30 €/km and 10 €/meal)	168.90	168.90	168.90	168.90	675.60	0	0
	Insurance	0	0	0	63.89	63.89	0	0

**Materials**	**Activities**							
	Office supplies and activities supplies (eg, cardboards, blue tack, sheet covers, dice)	2,526.20	2,526.20	2,526.20		7,578.60	1	7,578.60
	Food (eg, fruits, vegetables, tuna, bread, nuts, oil)	1,804.01	1,804.01	1,804.01		5,412.02	1	5,412.02
	Kitchen utensils (eg, spoon, knife, lunch box, fork, squeezer, plates, glasses)	223.97	223.97	223.97		671.91	1	671.91
	**Anthropometry**							
	Scales (900 €/unit), stadiometers (103 €/unit), flexible measuring tape (24.8 €/unit)		350.84	350.84	350.84	1052.52	0	0

**Additional didactic materials**	**Booklets**							
	Teachers and parents (3 €/book)	154.14	154.14	154.14		462.42	0	0
	Students (3 books/child; 3 €/book)	4,586.76	4,586.76	4,586.76		13,760.28	0	0
	**Workshop for parents**							
	(offices supplies and food)	0	1,579.92	0	0	1,579.92	0	0

**TOTAL COST**						**104,963.16**		**24,246.53**
**COST PER STUDENT**	**Intervention group (1,550)**					**54.13**		**15.64**

^a^EdAl: School-based intervention implemented in the academic years in the period 2007–2010 in Reus (Catalonia, Spain) based on lifestyle education.

The estimation of the per-child cost was calculated using the EdAl program implementation costs divided by the number of children who received the intervention and considering the baseline number of children in the intervention group.^11^

### Cost-effectiveness analysis of the EdAl program

The cost-effectiveness analysis outcomes were as follows:

The cost of the intervention for obesity-related outcomes was determined using the incremental cost-effectiveness ratio (ICER), which was defined as the difference in cost for the children in the intervention group and those in the control group divided by the difference in obesity-related measure effects between the intervention and control groups^11^: ICER = (Costs_Intervention Group_ − Costs_Control Group_)/(Effects_Intervention Group_ − Effects_Control Group_).

The ICER was calculated for four obesity-related outcomes: the number of obesity cases avoided, the decrease in obesity prevalence, the decrease in BMI units, and the decrease in BMI z-score units from the beginning to the end of the intervention. However, the cost-effectiveness analysis of the EdAl program was performed only for boys because the intervention was not effective for obesity-related outcomes for girls. The ICERs for obesity prevalence, BMI, and BMI z-scores were calculated by dividing the cost per child by the effects per child. For cases of avoided obesity, the ICER was evaluated by dividing the intervention cost (the cost per child multiplied by the total number of boys who received the intervention) by the number of obesity cases avoided among the boys in the total study population.

### Sensitivity of the cost-effectiveness analysis and scenario analysis

The main function of the sensitivity analysis was to evaluate the robustness of the result to possible variations.^12^ We calculated three scenario analyses: 1) including management costs; 2) including additional material, such as booklets and materials for parent workshops; and 3) including both (management and additional materials). The Euro value considered was in relation to the United States dollar in 2007 (when the EdAl program was implemented).

## RESULTS

### Cost analysis of the EdAl program results

Table 1 shows the costs of implementing the EdAl program for 1,550 7- and 8-year-old children, at baseline, in the 24 schools in the intervention group over 28 months (2.3 years) by 60 undergraduate students who were trained as HPAs. The cost of implementing the activities in the schools was obtained as follows: human resource costs for the HPAs training (10,584 €); materials for activities, such as office supplies (7,578.60 €); food (5,412.02 €); and additional didactic materials, such as kitchen utensils (671.91 €). Thus, the total cost was 24,246.53 € (total cost of the program) or 15.64 € per child or 5.21 €/child/year in the intervention group (for 1,550 children, including 805 boys and 745 girls).

### Cost-effectiveness analysis of the EdAl program results

After the 28-month intervention, the EdAl program yielded the following results for boys, stated as ICERs (Table 2): 968.66 € to avoid one case of obesity in boys (1.20 € per boy); 3.56 €/child to reduce the obesity prevalence by 1% in boys; 47.39 € for a decrease of one BMI unit per boy; and 65.17 € for a decrease of one BMI z-score unit per boy.

**Table 2.** Cost-effectiveness and cost-utility analysis in boys

	Intervention mean (bootstrapped 95% CI)	Control mean (bootstrapped 95% CI)	Mean difference (bootstrapped 95% CI)	ICER
**Obesity prevalence**	−2.36 (−4.52; −0.09)	2.03 (−0.59; 4.76)	−4,39 (−3,93; −4,85)	
** Total cost per participant (RCT)**	15,64 (14,52; 16,76)	0 €	15,64 €	3,56 (3,31; 3,82)
** Total cost per participant (scenario analysis 1)^a^**	19,57 (18,17; 20,97)	0 €	19,57 €	4,46 (4,14; 4,78)
** Total cost per participant (scenario analysis 2)^b^**	25,84 (23,99; 27,69)	0 €	25,84 €	5,89 (5,46; 6,31)
** Total cost per participant (scenario analysis 3)^c^**	29,77 (27,65; 31,86)	0 €	29,77 €	6,78 (6,3; 7,26)
**BMI z-score**	−0.05 (−0.07; −0.03)	0.19 (0.16; 0.21)	−0,24 (−0,23; −0,24)	
** Total cost per participant (RCT)**	15,64 (14,52; 16,76)	0 €	15,64 €	65,17 (60,5; 69,83)
** Total cost per participant (scenario analysis 1)^a^**	19,57 (18,17; 20,97)	0 €	19,57 €	81,54 (75,71; 87,38)
** Total cost per participant (scenario analysis 2)^b^**	25,84 (23,99; 27,69)	0 €	25,84 €	107,67 (99,96; 115,38)
** Total cost per participant (scenario analysis 3)^c^**	29,77 (27,65; 31,86)	0 €	29,77 €	124,04 (115,21; 132,75)
**BMI**	1.14 (0.92; 1.34)	1.47 (1.21; 1.72)	−0,33 (−0,2; −0,38)	
** Total cost per participant (RCT)**	15,64 (14,52; 16,76)	0 €	15,64 €	47,39 (44; 50,79)
** Total cost per participant (scenario analysis 1)^a^**	19,57 (18,17; 20,97)	0 €	19,57 €	59,3 (55,06; 63,55)
** Total cost per participant (scenario analysis 2)^b^**	25,84 (23,99; 27,69)	0 €	25,84 €	78,3 (72,7; 83,91)
** Total cost per participant (scenario analysis 3)^c^**	29,77 (27,65; 31,86)	0 €	29,77 €	90,21 (83,79; 96,55)

BMI, body mass index; ICER, incremental cost-effectiveness ratio; RCT, randomized controlled trial.

^a^including management costs.

^b^including additional material (booklets and materials for parents).

^c^including management + additional costs (booklets and materials for parents).

### Sensitivity analysis

The sensitivity analysis results are shown in Table 2. The ICER was calculated using three scenarios: 1) including management costs; 2) including additional material, such as booklets and materials for parent workshops; 3) including both (management and additional materials). The results differed according to whether obesity measurement, obesity prevalence, BMI, or BMI z-score was the outcome (Table 2). Moreover, as costs and effects were available at the individual level, a bootstrap approach was used to estimate the distribution of costs and health outcomes around the point estimates obtained in the actual study.

## DISCUSSION

The EdAl program, an intervention focusing on encouraging healthy lifestyle choices, such as diet and physical activity, to reduce obesity, is a cost-effective intervention for boys. In fact, the EdAl program costs 5.21 €/child/year (15.64 €/child in the intervention group over 28 months) to reduce the obesity prevalence in boys by 4.39%. As a result, it meets the Spanish Health Ministry’s 2009 criteria for cost-effectiveness: a cost of less than 5 € per child per year to achieve a greater than 2% reduction in the obesity prevalence.^13^ The EdAl program costs 2.4 €/child/year to reduce the obesity prevalence in boys by 2%. Moreover, the intervention could be considered cost-effective in all the scenarios that were analyzed; the reduction in obesity was greater than the minimum recommended by Spanish Health Ministry, and the cost did not exceed 10 € per child/year in all scenarios.^13^

Reductions in BMI and BMI z-score reflect a clear clinical impact for children, and these are the recommended obesity-related outcomes to consider.^14^ However, to the best of our knowledge, the clinical impact of reducing the obesity prevalence in a child population has not been determined.

The cost-effectiveness analysis of the EdAl program included obesity prevalence reduction as well as obesity-related outcomes, such as cases of avoided obesity, BMI, and BMI z-score reduction. In a published review, the cost to reduce BMI by one unit with preventive obesity interventions ranged from $1.16 (1.02 €) to $401 (355.7 €), which was lower than the cost of medical treatment or bariatric surgery ($1,000 or 887.14 €/BMI unit reduction).^3^ As our results and those of other school-based interventions demonstrate, prevention programs developed for and implemented in schools are more economical than programs implemented in health care centers or in the community by health professionals, such as the “Healthy Beginnings” health care intervention implemented in Sydney (Australia) and delivered to families to reduce childhood obesity.^15^

A school-based intervention study in China reported that a one-BMI-unit reduction in boys and girls over 12 months cost between 27 € and 313 €,^11^ a range that falls within the proposed intervals. The cost of the EdAl program, 47.39 € for one unit of BMI reduction per boy, falls at the lower end of this interval compared with the costs reported for other obesity prevention interventions.^11^^,^^16^ These results are in accordance with the review by Carter et al,^6^ which reported that interventions directed only toward children with obesity are less cost-effective than interventions that include all children, regardless of their BMI.

Moreover, the EdAl program decreased the BMI z-score by one unit at a cost of 65.16 € per boy over 28 months and prevented one case of obesity at a cost of 968.66 € (1.20 € per boy per 28 months). These results can be compared with a 1-year primary school-based intervention (children from 6 to 14 years old) in China, in which the cost to reduce the BMI z-score by one unit ranged from 58 € to 306 € and the cost of avoiding one case of obesity was approximately 1,308.9 € per student. Accordingly, the EdAl program appears to be more cost-effective than the school-based intervention implemented in China.^11^

In the present study, the most expensive category was HPA training, which represented 43% of the total EdAl program costs. This category should be excluded from calculations of the costs of sustained implementation because HPA training is currently part of the official curriculum (medicine and health sciences university education) at our university, so the university students must pay the course costs.^17^ As a result, HPA training provides an interesting opportunity to reduce the intervention costs associated with involving professionals in intervention implementation, which are typically high.^14^ Finally, implementing the intervention activities during school hours adds no costs related to location, electricity, or additional teaching hours.

The cost-effectiveness of the EdAl program could be analyzed only for boys because the obesity prevalence and obesity-related outcomes of girls were unchanged during this study.^5^ This gender difference in the obesity reduction response could be explained by physiological gender differences in this age group, particularly in terms of weight gained; the girls exhibited a consistent BMI over the course of the study.^18^ A review highlighted the different gender responses to school-based interventions in terms of anthropometric measurements and lifestyles.^19^ Collectively, the results indicate that interventions for children and adolescents may favor either boys or girls, depending on the age of participants, without providing a clear explanation for the differential effects.^19^ The obtained results have some implications for the implementation of the intervention in schools. If the intervention is to be implemented for both genders, changes will be needed for the intervention to achieve effective results for girls. Furthermore, 2 years after the cessation of the EdAl intervention, adolescents who participated in the EdAl program exhibited a stable obesity prevalence and improved healthy lifestyle practices.^20^ These results could support the long-term effectiveness of the program, but long-term cost-effectiveness should be assessed.

Some limitations of the EdAl program’s cost-effectiveness were identified. The foremost limitation is that, although there are existing cost-effectiveness frameworks,^21^ more evidence is necessary to determine a gold standard method for comparing the cost-effectiveness of school-based interventions.^22^ For instance, in Spain, assessments of the cost-effectiveness of school-based interventions implemented in recent years have been unable to identify the most cost-effective methods for preventing childhood obesity or to compare various school-based interventions.

The EdAl program is cost-effective for primary school-aged boys; thus, sustained implementation was only partially assessed because the majority of schools in Spain include both girls and boys. Although the EdAl program did not decrease the obesity prevalence in girls, some improvements in healthy lifestyles were observed. However, the EdAl program could implement intervention changes to reduce obesity-related outcomes in girls and achieve results similar to those achieved for boys.

In conclusion, the EdAl program is cost-effective for reducing the prevalence of obesity. A cost of 5.21 €/child/year reduced the prevalence of obesity in boys by 4.39%, which is half the cost that the Spanish Health Ministry established as cost-effective for the sustained implementation of obesity prevention programs. However, additional cost-effectiveness criteria should be defined.

## APPENDIX A. SUPPLEMENTARY DATA

eTable 1. CHEERS checklist
